# Supplementary material for: Integrative, segregative, and degenerate harmonics of the structural connectome
Source: Commun Biol. 2024 Aug 14;7:986. doi: 10.1038/s42003-024-06669-6 (PMC11324790; doi:10.1038/s42003-024-06669-6)
Supplement: Supplementary file 2 — Description of Additional Supplementary Materials [file 42003_2024_6669_MOESM2_ESM.pdf]

Description of Additional Supplementary Files

File name: Supplementary Data 1

Description: The source data and cognitive domain groupings for the Neurosynth analysis, described in the Methods section “Alignment with Meta-analytic Task Networks”

File name: Supplementary Data 2

Description: A table of exact statistics reported in the manuscript.
